# The trajectory of smoking cessation after treatment and its related factors in Taiwan

**DOI:** 10.1038/s41598-024-64311-1

**Published:** 2024-06-10

**Authors:** Chia-Hong Lin, Cing-Ya Wang, Kuan-Fen Chen, Shu-Pi Chiu, Wan-Ting Huang, Sheng-Yu Fan

**Affiliations:** 1 Department of Family Medicine, Ditmanson Medical Foundation Chia-Yi Christian Hospital, Chia-Yi, Taiwan; 2 Community Nursing Room, Ditmanson Medical Foundation Chia-Yi Christian Hospital, Chia-Yi, Taiwan; 3 Clinical Medicine Research Center, Ditmanson Medical Foundation Chia-Yi Christian Hospital, Chia-Yi, Taiwan; 4 Institute of Gerontology, College of Medicine, National Cheng Kung University, No. 1 University Road, Tainan, 701 Taiwan

**Keywords:** Cigarette dependence, Longitudinal study, Smoking cessation, Trajectory, Human behaviour, Risk factors, Lifestyle modification

## Abstract

Smoking has multiple negative effects on health; therefore, the Taiwanese government provides smoking cessation clinics to smokers. This study aimed to explore the trajectory of smoking cessation after smokers received treatment and the variables related to different trajectories. A retrospective longitudinal study was conducted, in which 735 adult smokers who received smoking cessation medications were recruited. The participants’ demographic characteristics, chronic diseases, smoking characteristics, and cigarette dependence were collected from chart review. The amount of smoking was collected at baseline, and at 1 week, 1 month, 3 months, and 6 months after treatment. The Proc Traj procedure for group-based modeling and multinomial logistic regression were used for statistical analysis. Three trajectories were identified: early quitters (28.03%), late quitters (11.43%) and reducers (60.54%). Compared with early quitters, reducers were younger and had a higher probability of severe cigarette dependence. Compared with early quitters, late quitters had a higher number of taking smoking cessation medications. The findings revealed that approximately 60% of participants who received smoking cessation treatment could not completely quit smoking, and that age, number of medications taken, and cigarette dependence were significant predictors of different trajectories.

## Introduction

Smoking has multiple negative effects on health, including shortening life expectancy and disability-free life expectancy, increasing the number of years lived with a disability, and decreasing health-related quality of life^1,2^. In 2020, the smoking rate in Taiwan was 13.1% (men: 23.1%, women: 2.9%), with the highest smoking rate in men among those 46–50 years old (39.7%) and women aged among those 21–25 years old (7.5%)^3^. To mitigate the impact of smoking, the Taiwanese government has deployed multiple strategies, not only policy-based but also personal approaches, one of which is the establishment of smoking cessation clinics^4^.

Smoking is an easy relapse behavior, with studies showing smoking relapse in 37.1% of British^5^ and 69.5% of Taiwanese^6^ respondents after the initial 1 year of smoking abstinence. Previous studies have focused on the developmental trajectories of smoking in adolescents^7^, young adults^8^, and adults throughout their lifespan^9,10^, showing changes in smoking status. A study with a 1-year follow-up found that 72% of smokers without quit motivation had a stable trajectory in smoking^11^. Except for stable nonsmokers or smokers, certain groups of people increased, decreased, or quit smoking.

Some studies have explored smoking trajectories following smoking cessation medication intervention. In a survey of 300 female smokers, after using a nicotine patch for 1 month, the participants were divided into three groups: abstainers who did not smoke in the first week and first month (132, 44.0%), early lapsers who smoked in the first week (75, 25.0%), and late lapsers (93, 31.0%)^12^. In another study of 265 adolescents, after six months of nicotine replacement treatment, the participants showed three trajectories: persistent smokers (34.6%), moderate decreasers (16.0%), and stronger decreasers (49.6%)^13^. A proportion of smokers who received smoking cessation medication would still smoke or relapse.

Free smoking cessation medication combining sustained telephone counseling could improve the smoking quit rate^14^. The Taiwan government has implemented a smoking cessation policy, providing individuals over aged 18 and above who smoke with access to free medications at smoking cessation clinics. Smoking cessation case managers follow up smoking status by telephone to improve the outcome of smoking cessation.

Regarding variables related to different trajectories, men^10,15^, younger age^15,16^, low educational level^17^, lower socioeconomic status, unemployment^10^, low-skill work^9^, and blue-collar workers^18^ tended to have worse trajectories, including relapse, increased smoking, or persistent smoking. Smokers with chronic diseases had more smoking cessation attempts and were more likely to quit smoking than those without^19,20^. In addition, previous smoking volume^12,17,21–23^, years of smoking^16^, previous attempts to quit^11^, intention to quit^11,13,15–17^, and cigarette dependence^15,21,23–25^ were significantly associated with smoking trajectories.

Smokers may not quit smoking in the early stage of taking medication, and they may relapse after a period of time. Identifying the trajectories of smoking cessation and related variables are useful for clinical care for essential interventions. This study aimed to explore the trajectories of smoking cessation among patients in a smoking cessation clinic and variables related to different trajectories.

## Methods

### Study design

This was a retrospective longitudinal study. Smoking cessation case managers approached potential patients who smoked. When the patients were willing to join the smoking cessation program, they were referred to the smoking cessation clinics. They could obtain smoking cessation mediations from the pharmacy department of the hospital. Typically, they would get 1 weeks’ supply each time and could return to the clinics if needed. The medications were provided free of charge. Care managers conducted follow-ups at four timepoints: at the beginning of treatment (T1), after 1 week (T2), 3 months (T3), and 6 months (T4). The care managers collected T1 data face-to-face and T2 to T4 data via telephone. The data were recorded in the charts.

The research team obtained ethical approval from the institutional review board of the Ditmanson Medical Foundation of, Chia-Yi Christian Hospital (IRB number: IRB2021067) and then collected data from the charts. The data period spanned from January 2019 to December 2019. All participants recruited for the face-to-face survey signed informed consent forms. All procedures were performed in accordance with the principles of the Declaration of Helsinki’s statement.

### Participants

The participants included: (1) smokers aged over 18 years old, (2) those willing to attend the smoking cessation clinics to quit smoking, and (3) those receiving smoking cessation medication at a smoking cessation clinic located in a district teaching hospital in Southern Taiwan.

### Measurements

The demographic characteristics included age, sex, educational level, and work status. Work included none, blue-collar workers (construction, manufacturing, transportation, agriculture, forestry, fisheries, and animal husbandry), and white-collar workers (government employees, financial, service, medical, and commercial).

Chronic diseases included whether the participants had cancer, lung disease, heart disease, cerebrovascular disease, hypertension, diabetes, liver disease, kidney disease, hyperlipidemia, and others (yes or no). The total number of chronic diseases was calculated.

Smoking characteristics included years of smoking, number of cigarettes smoked a day before treatment, and cigarette addiction, which were assessed using the Fagerstrom Tolerance Questionnaire (FTQ)^26,27^. The FTQ includes six items, ranging from 0 to 10: 0 to 5 being mild dependence, 6 to 7 moderate dependence, and 8 to 10 severe dependence. The translated Taiwanese version of the FTQ has shown good reliability and validity^28^. The number of individuals taking smoking cessation medication was also collected.

A three-point Likert scale was used to evaluate the amount of smoking: “Compared with the amount of smoking before treatment, how many cigarettes do you smoke in seven days?” (3 = none; 2 = less than before; 1 = the same).

### Statistical analysis

Descriptive analysis was used to present demographic, chronic disease, and smoking characteristics and group-based trajectory modeling was used to identify the trajectory patterns of smoking cessation^29^. Univariate analysis was used to test the differences in the predictors between the different types of trajectories, and multinomial logistic regression was used to identify the variables related to the different trajectories. The dependent variables were the different trajectory types, and significant variables in the univariate analysis were entered as predictors. The Bonferroni correction was used to reduce the inflated type I error rate^30^. As there were five predictors (age, number taking smoking cessation medication, number of cigarettes smoked per day, and moderate and severe cigarette dependence), a significant level of p less than 0.01 (0.05/5) was utilized.

Regarding the estimation of sample size, at least 500 participants were required for group-based trajectory modeling^31^, with each group necessitating at least 5% of the total participants^32^. The study’s sample size met group-based trajectory modeling and multinomial logistic regression. Statistical analyses were conducted using SAS version 9.0. This study adhered to the Strengthening the Reporting of Observational Studies in Epidemiology (STROBE) guidelines^33^.

## Results

In total, 735 participants enrolled in this study. Their mean age was 52.82 years old (*SD* = 13.23); 89.25% were men, 40.14% completed senior high school education, and 40.27% were blue-collar workers. Regarding chronic diseases, 59.46% of participants reported having chronic conditions, with hypertension (31.97%) being the most prevalent. The average duration of smoking was 30.14 years (*SD* = 12.61), and participants smoked an average of 25.37 cigarettes per day (*SD* = 14.30). Mild, moderate, and severe cigarette dependence were reported by 19.86%, 34.29%, and 45.85% of participants, respectively (see Table 1). All participants received smoking cessation treatment, with 725 (98.64%) prescribed Champix and 10 (1.36%) receiving Nicotinell TTS.

**Table 1:** Participant characteristics.

Variables	All participants (N = 735)	Reducers (N = 445)	Late quitters (N = 84)	Early quitters (N = 206)	*t* or χ^2^/*p* value
Age	52.82 (13.23)	51.97 (13.42)	52.26 (13.92)	54.89 (12.31)	3.54, 0.029
Sex					
Male	656 (89.25%)	402 (90.34%)	74 (88.10%)	180 (87.38%)	1.42, 0.492
Female	79 (10.75%)	43 (9.66%)	10 (11.90%)	26 (12.62%)	
Educational level					
Elementary school	146 (19.86%)	81 (18.20%)	18 (21.43%)	47 (22.82%)	
Junior high school	173 (23.54%)	109 (24.49%)	22 (26.19%)	42 (20.39%)	
Senior high school	295 (40.14%)	180 (40.45%)	31 (36.90%)	84 (40.78%)	
Undergraduate and above	121 (16.46%)	75 (16.85%)	13 (15.48%)	33 (16.02%)	
Work					
None	210 (28.57%)	119 (26.74%)	24 (28.57%)	67 (32.52%)	2.83, 0.586
White-collar workers	229 (31.16%)	143 (32.13%)	28 (33.33%)	57 (27.67%)	
Blue-collar workers	296 (40.27%)	181 (40.67%)	32 (38.10%)	82 (39.81%)	
Chronic disease					
Cancer	20 (2.72%)	11 (2.47%)	6 (7.14%)	3 (1.46%)	5.93, 0.052
Lung diseases	31 (4.22%)	24 (5.39%)	3 (3.57%)	4 (1.94%)	4.77, 0.092
Heart diseases	72 (9.80%)	43 (9.66%)	7 (8.33%)	22 (10.68%)	0.39, 0.821
Cerebrovascular diseases	24 (3.27%)	16 (3.60%)	4 (4.76%)	4 (1.94%)	2.01, 0.367
Hypertension	235 (31.97%)	133 (29.89%)	28 (33.33%)	74 (35.92%)	2.44, 0.295
Diabetes	125 (17.01%)	79 (17.75%)	14 (16.67%)	32 (15.53%)	0.50, 0.779
Liver diseases	53 (7.21%)	39 (8.76%)	5 (5.95%)	9 (4.37%)	4.29, 0.117
Kidney diseases	15 (2.04%)	10 (2.25%)	4 (4.76%)	1 (0.49%)	5.95, 0.051
Hyperlipidemia	49 (6.67%)	27 (6.07%)	9 (10.71%)	13 (6.31%)	2.51, 0.285
Other	160 (21.77%)	102 (22.92%)	21 (25.00%)	37 (17.96%)	2.62, 0.270
Number of chronic diseases	1.07 (1.15, 0–7)	1.09 (1.16)	1.20 (1.35)	0.97 (1.02)	1.45, 0.234
Years of smoking	30.14 (12.61)	29.79 (12.61)	30.08 (13.63)	30.92 (12.61)	0.57, 0.565
Number of cessation medication usage	1.48 (0.71)	1.50 (0.72)	1.73 (0.77)	1.36 (0.64)	6.60, 0.001
Daily cigarettes consumption	25.37 (14.30)	27.09 (13.95)	21.92 (16.03)	23.07 (13.77)	8.52, < 0.001
Cigarette dependence					
Mild dependence	146 (19.86%)	65 (14.61%)	25 (29.76%)	56 (27.18%)	36.78, < 0.001
Moderate dependence	252 (34.29%)	139 (31.23%)	29 (34.52%)	84 (40.78%)	
Severe dependence	337 (45.85%)	241 (54.16%)	30 (35.72%)	66 (32.04%)	

**Figure 1:**
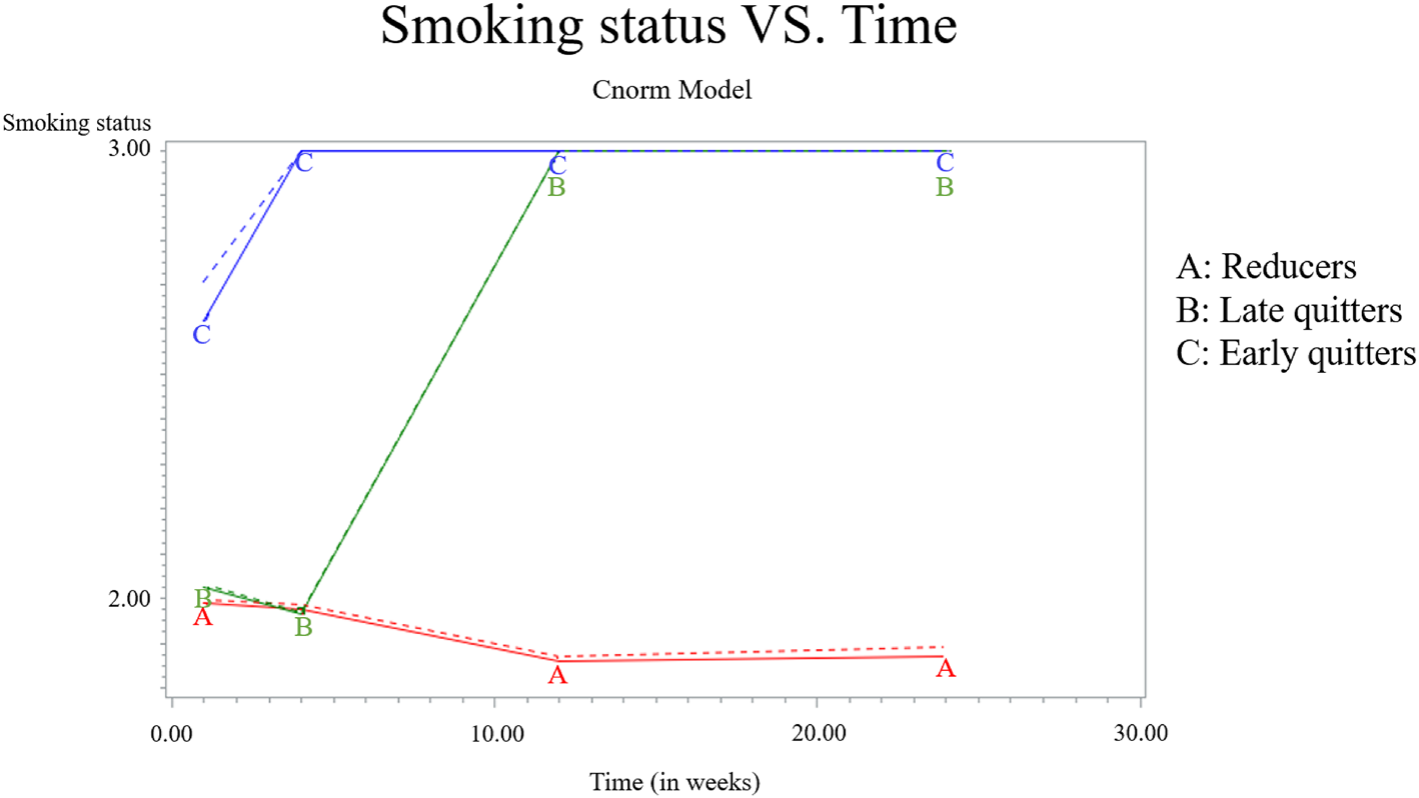
Smoking cessation trajectories. (Smoking status: 3 = Participants abstained from smoking for 7 consecutive days, 2 = Participants reduced smoking compared to previous levels for 7 days. Timing of data collection: T1 = at the beginning of treatment, T2 = after 1 week, T3 = 3 months, T4 = 6 months).

Three smoking cessation trajectories were identified (refer to Fig. 1). Trajectory 1 comprised “reducers” (*n* = 445, 60.54%), representing participants who smoked less than before the treatment across all periods. Trajectory 2 were “late quitters” (*n* = 84, 11.43%), reflecting participants who smoked less than before at T2 and abstained from smoking at T3 and T4. Trajectory 3 were “early quitters” (*n* = 206, 28.03%), comprising participants who refrained from smoking at T2, T3, and T4.

The univariate analysis revealed differences among the three groups in age (*F* = 3.54, *p* = 0.029), number of smoking cessation medications taken (*F* = 6.60, *p* = 0.001), number of cigarettes smoked per day (*F* = 8.52, *p* < 0.001), and cigarette dependence (*χ*^*2*^ = 36.78, *p* < 0.001). Reducers were younger than early quitters, smoked more cigarettes per day than early and late quitters, and had more severe cigarette dependence. Late quitters had a higher number of smoking cessation medications taken than early quitters (see Table 1). Furthermore, compared with early quitters, reducers were younger (*B* =  − 0.02, *p* = 0.007) and had a higher probability of severe cigarette dependence (*B* = 1.26, *p* < 0.001). Late quitters had a higher number of smoking cessation medications taken than early quitters (*B* = 0.73, *p* < 0.001) (see Table 2).

**Table 2:** Results of multinomial logistic regression analysis.

	Reducers			Late quitters		
	B	Exp (B) (95 CI)	*p*	B	Exp (B) (95 CI)	*p*
Age	− 0.02	0.98 (0.97, 1.00)	0.007	− 0.02	1.00 (0.98, 1.02)	0.858
Frequency of cessation medication usage	0.24	1.27 (0.95, 1.70)	0.112	0.73	2.07 (1.39, 3.08)	< 0.001
Daily cigarettes consumption	− 0.01	1.00 (0.98, 1.01)	0.538	− 0.01	0.99 (0.96, 1.01)	0.319
Cigarette dependence						
Mild dependence	REF	REF	REF	REF	REF	REF
Moderate dependence	0.77	2.16 (1.18, 3.96)	0.013	− 0.33	0.72 (0.33, 1.60)	0.421
Severe dependence	1.26	3.52 (1.82, 6.84)	< 0.001	− 0.21	0.65 (0.32, 2.03)	0.650

Reference group: Early quitters.

## Discussion

This study used longitudinal data to explore the smoking trajectories among participants who received smoking cessation medications. Three trajectories were identified: early quitters, late quitters, and reducers. The significantly related variables included age, number of taking smoking cessation medications, and cigarette dependence.

Early quitters stopped smoking at the start of the intervention and maintained until 6 months later. Late quitters decreased their smoking from the beginning to 3 months and then stopped smoking until 6 months. Meanwhile, reducers continued smoking throughout the entire period but smoked less than before. Previous studies have demonstrated success rates of quitting ranged from 16^17^ to 26.9^13^ to 44%^12^. Previous studies showed one of trajectories that more than 30% of the participants were persistent smokers^13^, and there were early and late relapse trajectories^12^. The success rate in this study, including early and late quitters, was 39.46%. Notably, smoking cessation medications were used in this study, whereas Cofta-Woerpel et al.^12^ used nicotine replacement therapy. Medication success rates may be higher than psychosocial interventions^16,17^.

Like previous studies^15,16^, the older participants in this study tended to quit smoking. On the other hand, a four-country survey showed younger smokers had higher attempts to quit and were more likely to quit than older smokers. However, after adjustment for nicotine dependence, the relationship between age and smoking abstinence was insignificant. Heaviness of smoking may play a more important role in smoking cessation^34^. A 4-year longitudinal study focusing on middle-aged and older adults showed smoking cessation related to older adults and poor self-rated health and smoking relapse related to younger age and good self-rated health^35^. Age may interrelate with smoking years, health, and nicotine dependence^34^. Most participants in this study were middle-aged adults, and older people may be more concerned about their health and attempt to quit smoking.

The results support cigarette dependence as a predictor of smoking cessation, which is consistent with previous studies^15,21,23–25^; however, differences in cigarette dependence only occurred between early quitters and reducers. Nicotine dependence has not only a biological mechanism but also a psychological mechanism on perceived behavioral control related smoking cessation^36^. Smokers with higher nicotine dependence had more difficulty in smoking cessation.

Adherence to smoking cessation medications was significantly related to successful smoking cessation^37,38^. Early quitters and late quitters had a similar process from 3 to 6 months, and late quitters took more cessation medication than early quitters. The potential reason was that late quitters had higher needs for medications, which were helpful for the reduction of smoking from 3 to 6 months.

Regarding clinical implications, healthcare professionals could focus their attention on monitoring the amount of smoking 1 month after treatment to determine the likelihood of participants achieving smoking cessation. Notably, approximately 60% of the participants continued smoking. Thus, medication alone is not sufficient for smoking cessation, and other psychosocial interventions may be needed^39^.

The strength of this study is its utilization of longitudinal data from a large sample size. However, there were some limitations. First, smoking status relied on self-reports rather than objective physical measurements such as carbon monoxide levels. Additionally, using a three-point Likert scale may not capture nuances compared with detailed levels of smoking assessment. Second, participants were recruited from outpatient clinics of a single hospital, comprising individuals willing to quit smoking and open to smoking cessation medications, with no collection of data on medication adherence. The results may not be generalized to other populations. Future studies should collect physical measurements over longer follow-up periods.

In conclusion, this study identified three trajectories following the administration of smoking cessation medication: early quitters, late quitters, and reducers. It was observed that participants who were older, adhered to smoking cessation medications, and exhibited lower levels of cigarette dependence tended to be early quitters.

## Data Availability

Data supporting the findings of this study are available upon request from the corresponding author. The data are not publicly available due to ethical restrictions.
